# The Enigmatic Origin of Bovine mtDNA Haplogroup R: Sporadic Interbreeding or an Independent Event of *Bos primigenius* Domestication in Italy?

**DOI:** 10.1371/journal.pone.0015760

**Published:** 2010-12-28

**Authors:** Silvia Bonfiglio, Alessandro Achilli, Anna Olivieri, Riccardo Negrini, Licia Colli, Luigi Liotta, Paolo Ajmone-Marsan, Antonio Torroni, Luca Ferretti

**Affiliations:** 1 Dipartimento di Genetica e Microbiologia, Università di Pavia, Pavia, Italy; 2 Dipartimento di Biologia Cellulare e Ambientale, Università di Perugia, Perugia, Italy; 3 Istituto di Zootecnica, Università Cattolica del Sacro Cuore, Piacenza, Italy; 4 Dipartimento di Morfologia, Biochimica, Fisiologia e Produzioni Animali, Università di Messina, Messina, Italy; University of Cambridge, United Kingdom

## Abstract

**Background:**

When domestic taurine cattle diffused from the Fertile Crescent, local wild aurochsen (*Bos primigenius*) were still numerous. Moreover, aurochsen and introduced cattle often coexisted for millennia, thus providing potential conditions not only for spontaneous interbreeding, but also for pastoralists to create secondary domestication centers involving local aurochs populations. Recent mitochondrial genomes analyses revealed that not all modern taurine mtDNAs belong to the shallow macro-haplogroup T of Near Eastern origin, as demonstrated by the detection of three branches (P, Q and R) radiating prior to the T node in the bovine phylogeny. These uncommon haplogroups represent excellent tools to evaluate if sporadic interbreeding or even additional events of cattle domestication occurred.

**Methodology:**

The survey of the mitochondrial DNA (mtDNA) control-region variation of 1,747 bovine samples (1,128 new and 619 from previous studies) belonging to 37 European breeds allowed the identification of 16 novel non-T mtDNAs, which after complete genome sequencing were confirmed as members of haplogroups Q and R. These mtDNAs were then integrated in a phylogenetic tree encompassing all available P, Q and R complete mtDNA sequences.

**Conclusions:**

Phylogenetic analyses of 28 mitochondrial genomes belonging to haplogroups P (N = 2), Q (N = 16) and R (N = 10) together with an extensive survey of all previously published mtDNA datasets revealed major similarities between haplogroups Q and T. Therefore, Q most likely represents an additional minor lineage domesticated in the Near East together with the founders of the T subhaplogroups. Whereas, haplogroup R is found, at least for the moment, only in Italy and nowhere else, either in modern or ancient samples, thus supporting an origin from European aurochsen. Haplogroup R could have been acquired through sporadic interbreeding of wild and domestic animals, but our data do not rule out the possibility of a local and secondary event of *B. primigenius* domestication in Italy.

## Introduction

The domestication of wild aurochs (*Bos primigenius*) ∼10 thousand years ago (kya) was a major development in the Neolithic transition, with significant cultural and socioeconomic implications for the numerous human populations of the Old World that at different times adopted cattle breeding [1], [2]. However, despite the wide geographical distributions of *B. primigenius* (Asia, Europe and North Africa), archeozoological and genetic evidence, mainly from mitochondrial DNA (mtDNA), suggest that modern cattle might represent the legacy of just two independent and geographically distinct domestication events, both of which occurred in southwest Asia. One occurred in the Fertile Crescent and involved aurochsen characterized by mtDNAs belonging to the macro-haplogroup T, whose different sub-branches (T1, T2, T3, T4 and T5) characterize modern taurine breeds (*Bos taurus*). The other, which occurred most likely in the Indus Valley, led to the humped zebuine breeds (*Bos indicus*) and involved *B. primigenius* population(s) with mtDNAs belonging to haplogroups I1 and I2 [3]–[12].

The T sub-haplogroups are rather geographically structured: T1 is the most common in Africa, T3 is dominant in Europe, while T4 is typical of East Asian breeds. All T mtDNA clusters (except T4) are found in Near Eastern breeds [5], [13], [14]. This, together with the divergence time of the entire macro-haplogroup T, only 16.0 ky obtained by using a constant mtDNA sequence evolution rate and an estimated bifurcation between yak and bison versus cattle of 2 million years [9], agrees with a single *B. taurus* domestication event in the Fertile Crescent, followed by spread with the migrations of farmers and pastoralists. However, note that much deeper divergence times for all *Bos* lineages have been extrapolated from the phylogenetic relationships of artiodactyl and cetacean mitogenomes using a fixed bifurcation time of 60 million years between ruminants and whales [15].

When domesticated herds diffused from the Fertile Crescent into Europe, Africa and the rest of Asia, local *B. primigenius* populations were numerous and widespread. Moreover, the coexistence of autochthonous wild aurochsen and the newly introduced cattle lasted for thousands of years in many geographical areas, thus providing potential conditions not only for spontaneous interbreeding between wild animals and domestic herds, but also for pastoralists to create secondary centers of domestication involving local aurochs populations. Additional domestication events – in Africa [16]–[18], East Asia [13] and possibly southern Europe [19] – have been proposed, but they are challenged by the age estimates obtained from a large dataset of entire mtDNA sequence data [9]. Indeed, the supposedly involved haplogroups (T1 for Africa, T4 for East Asia and T3 for Europe) are all subsets of T, which is 16.0±3.2 ky old according to Achilli et al. 2008 [9]. The domestication of these closely related sub-haplogroups of T independently from geographically distinct *B. primigenius* populations, would imply that, despite the great distances, aurochs populations from northern Africa, southern Europe and East Asia shared and maintained an almost complete homogeneity at the level of the entire mitochondrial sequence. This would be an unprecedented scenario for Eurasian mammals [20]–[23] that nevertheless has recently received some support from the identification of mtDNA control-region sequences in pre-Neolithic aurochs remains from Italy that appear to belong to the macro-haplogroup T, mostly T3 [24].

The debate concerning the origin of domesticated cattle has received new fuel from the finding that not all taurine mtDNAs belong to haplogroups T1–T5. The analysis of entire mitochondrial genomes from modern taurine cattle has indeed detected representatives of three mtDNA branches radiating prior to the T node in the bovine phylogeny. The first is haplogroup Q. Out of the three non-T branches, this is the closest to haplogroup T, with a divergence time of about 48 ky [9], [10]. The second is haplogroup P, found in an animal from Korea generically classified as “beef cattle” [9]. Its estimated divergence time from haplogroup T is about 71 ky [10], in agreement with the scenario that P was a marker of European aurochs, especially those from northern and central Europe [24]–[27]. The third is haplogroup R, encompassing so far only four mtDNAs, all from Italian taurine breeds. The separation between R and the other taurine branches represents the earliest known split in the mtDNA phylogeny of *B. primigenius* [10].

The identification and the analysis of these few non-T mtDNAs has not only revealed that *B. primigenius* populations of Western Eurasia harbored extensive variation at the level of mitochondrial genomes, but also that such a diversity was probably geographically well-structured. Thus, haplogroups P, Q and R represent excellent tools to properly evaluate the possibility that independent events of cattle domestication, other than those in southwest Asia, have occurred. Such an assessment requires the identification of an adequate number of non-T mtDNAs and a detailed analysis of their sequence variation.

To this aim, we performed a comprehensive mtDNA survey of more than 1,700 animals belonging to a number of European breeds. We detected ten novel Q and six novel R mtDNAs, which were completely sequenced. Our data support the scenario that haplogroup Q spread from the Middle East together with haplogroups T1, T2, T3, T4 and T5, but raise the possibility of an Italian origin for haplogroup R.

## Results

### Survey of mtDNA Haplogroups in European Taurine Breeds

The mtDNA control region (CTR), approximately from np 15823 to 215, was sequenced in 1,747 animals representing 37 European breeds, of which 25 were Italian and the remaining twelve from other European regions. This dataset comprises 1,128 new subjects and 619 from a previous study [10]. The control-region mutational motifs allowed the classification of virtually all the mtDNAs within known haplogroups as shown in Table 1. The complete list of CTR haplotypes is available in Table S1. Concerning the overall haplogroup frequencies, T3 (mutational motif: BRS) was the most frequent (88.6%), as expected from a survey of European cattle; T1 (motif: 169-16113-16255) and T2 (motif: 169-16057C-16185-16255) had similar low frequencies, 4.2% and 5.0%, respectively. We did not detect any mtDNA belonging to haplogroup T4 (motif: 169-16042-16093-16302), while twelve mtDNAs (0.7%) with the rare haplogroup T5 mutational motif (163-169-16255) were identified. Most of the T5 mtDNAs were found in the Valdostana breed where this haplogroup appears to be common (13.5%). Only two mtDNAs (0.1%) from an Agerolese and a Cabannina did not cluster into any of the T subclades, but as previously reported they belonged to haplogroup T1′2′3 (motif: 169-16255) [10].

**Table 1:** Frequencies of MtDNA haplogroups in european cattle breeds.

Breed	No.of	Haplogroup (%)						
	subjects	T1′2′3	T1	T2	T3	T5	Q	R
Agerolese	33	3.0	12.2	-	81.8	-	-	3.0
Bianca Val Padana	4	-	-	-	100.0	-	-	-
Blacksided Trondheim	5	-	-	-	100.0	-	-	-
Bulgarian Grey	30	-	-	23.3	76.7	-	-	-
Burlina	1	-	-	-	100.0	-	-	-
Cabannina	38	2.6	-	2.6	92.2	-	2.6	-
Calvana	25	-	8.0	-	92.0	-	-	-
Chianina	290	-	6.6	8.3	83.4	-	1.7	-
Cinisara	63	-	15.9	1.6	79.4	-	-	3.2
Eastern Finn Cattle	7	-	-	-	100.0	-	-	-
Garfagnina	2	-	-	50.0	50.0	-	-	-
Grey Alpine	45	-	-	4.4	91.2	-	4.4	-
Grey Steppe	18	-	5.6	11.2	83.2	-	-	-
Holstein	2	-	-	-	100.0	-	-	-
Italian Brown	9	-	11.1	-	88.9	-	-	-
Italian Friesian	186	-	-	0.5	97.3	2.2	-	-
Italian Podolian	76	-	10.5	3.9	85.6	-	-	-
Italian Red Pied	125	-	0.8	8.0	89.6	0.8	0.8	-
Jersey	18	-	-	-	100.0	-	-	-
Limousine	50	-	6.0	2.0	92.0	-	-	-
Marchigiana	139	-	7.9	3.6	87.8	-	-	0.7
Maremmana	22	-	13.6	13.6	72.8	-	-	-
Modicana	12	-	-	-	100.0	-	-	-
Mucca Pisana	33	-	-	-	100.0	-	-	-
Ottonese	7	-	-	-	100.0	-	-	-
Pettiazza	35	-	-	-	100.0	-	-	-
Piedmontese	70	-	-	2.9	97.1	-	-	-
Reggiana	38	-	5.3	-	94.7	-	-	-
Rendena	1	-	-	-	100.0	-	-	-
Romagnola	222	-	4.1	8.6	82.4	-	2.3	2.7
Savoiarda	2	-	-	-	100.0	-	-	-
Simmental	9	-	-	-	100.0	-	-	-
Swedish Red Polled	10	-	-	10.0	90.0	-	-	-
Swiss Brown	1	-	-	-	100.0	-	-	-
Telemark	5	-	-	-	100.0	-	-	-
Valdostana	52	-	-	1.9	84.6	13.5	-	-
Vestland Red Polled	5	-	-	-	100.0	-	-	-
Unknown a	57	-	-	5.3	94.7	-	-	-
***Total***	1747	0.1	4.2	5.0	88.6	0.7	0.8	0.6

a European cattle for which a specific breed affiliation was not available.

Although mtDNAs belonging to the T branch were by far the most frequent (98.6%), fourteen animals (0.8%) harbored mtDNAs with the control-region motif of the Q haplogroup (169-15953G-16255) and ten (0.6%) were characterized by the haplogroup R motif (8-106-166-221+C-234+T-249-296-300-15818-15900-15951-15953G-16057-16076-16084-16085-16121-16122-16127-16135-16137-16200+A-16231-16248-16250-16264-16301). None of the mtDNAs fell within haplogroup P (motif: 106-166-190-221+C-222-249-300-301-15951-15953G-15994-16049-16051-16058-16074-16085-16122-16231-16255-16264).

### Phylogenetic Relationships of the non-T mtDNAs

**Figure 1:**
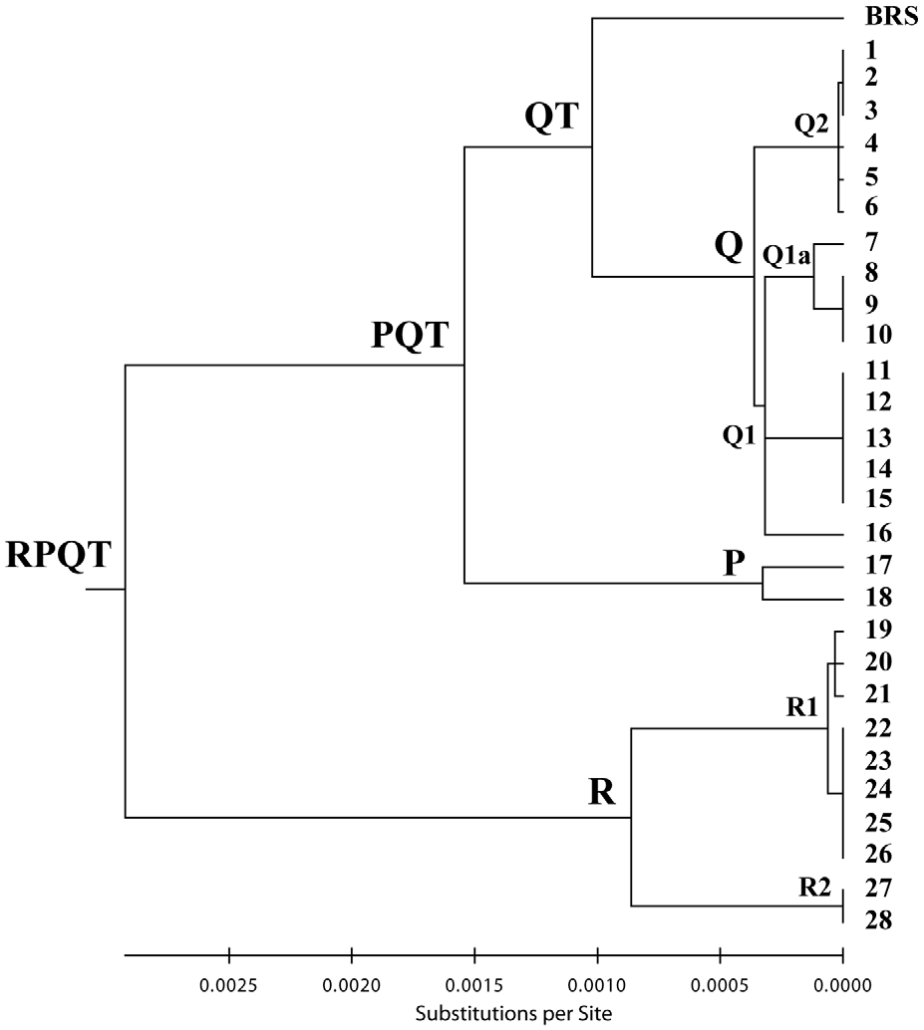
Most-parsimonious tree of bovine complete MtDNA sequences. The tree is drawn to scale. Phylogeny construction was performed by hand following a parsimony approach, while the evolutionary distances were computed using the Maximum Likelihood (ML) method. The exact values for clades and subclades are available in Table 3 together with averaged distance (rho) of the haplotypes of a clade to the respective root haplotype. Sixteen of the sequences (#1–3, #5, #9–13, #16, #22–26, #28) are new, while the others were previously published: *Bos taurus* Reference Sequence (BRS) (GenBank accession number V00654) [45] # 14–15, #17 [9]; #4, #6–8, #19–21, #27 [10]; #18 [27]. Taking into account that sequence #18 is from an ancient British aurochs radiocarbon dated to 6,738±68 calibrated years BP, the divergence of the haplogroup P node is underestimated. Additional information regarding each mtDNA sequence, including GenBank accession numbers, is provided in Table 2. An additional branch, named E, placed between P and R has been reported previously [10], [26] but was not included in the tree since it is not a complete mitochrondrial genome sequence.

The 16 newly discovered non-T mtDNAs were sequenced completely and their phylogenetic relationships are described in Figure 1, together with the ten previously reported Q and R sequences, as well as two P sequences [9], [10], [27]. Sequencing of the entire molecule confirmed that all non-T mtDNAs were indeed members of haplogroups Q and R. The list of complete MtDNA sequences belonging to haplogroups P, Q and R is shown in Table 2.

**Table 2:** List of complete MtDNA sequences belonging to haplogroups P, Q and R.

ID#	Sample ID	Haplogroup	Breed	GenBank ID	Reference
1	CHI 467	Q2	Chianina	HQ184030	This study
2	CHI 490	Q2	Chianina	HQ184031	This study
3	CHI 597	Q2	Chianina	HQ184032	This study
4	ROM 445	Q2	Romagnola	FJ971080	Achilli et al. 2009
5	ROM 550	Q2	Romagnola	HQ184033	This study
6	CHI 413	Q2	Chianina	FJ971081	Achilli et al. 2009
7	PRI 19	Q1a	Italian Red Pied	FJ971082	Achilli et al. 2009
8	ROM 475	Q1a	Romagnola	FJ971083	Achilli et al. 2009
9	ROM 534	Q1a	Romagnola	HQ184034	This study
10	ROM 590	Q1a	Romagnola	HQ184035	This study
11	GAL 5	Q1	Grey Alpine	HQ184036	This study
12	GA 16	Q1	Grey Alpine	HQ184037	This study
13	CAB 1	Q1	Cabannina	HQ184038	This study
14	CAB 2	Q1	Cabannina	EU177866	Achilli et al. 2008
15	CAB 3	Q1	Cabannina	EU177867	Achilli et al. 2008
16	CHI 466	Q1	Chianina	HQ184039	This study
17	FC3	P	Korean beef cattle	DQ124389	Achilli et al. 2008
18	CPC98	P	*Bos primigenius*	GU985279	Edwards et al. 2010
19	PER 12	R1	Agerolese	FJ971084	Achilli et al. 2009
20	CIN 13	R1	Cinisara	FJ971085	Achilli et al. 2009
21	CIN 19	R1	Cinisara	FJ971086	Achilli et al. 2009
22	ROM 498	R1	Romagnola	HQ184040	This study
23	ROM 553	R1	Romagnola	HQ184041	This study
24	ROM 561	R1	Romagnola	HQ184042	This study
25	ROM 584	R1	Romagnola	HQ184043	This study
26	ROM 600	R1	Romagnola	HQ184044	This study
27	ROM 478	R2	Romagnola	FJ971087	Achilli et al. 2009
28	MCG 375	R2	Marchigiana	HQ184045	This study

**Figure 2:**
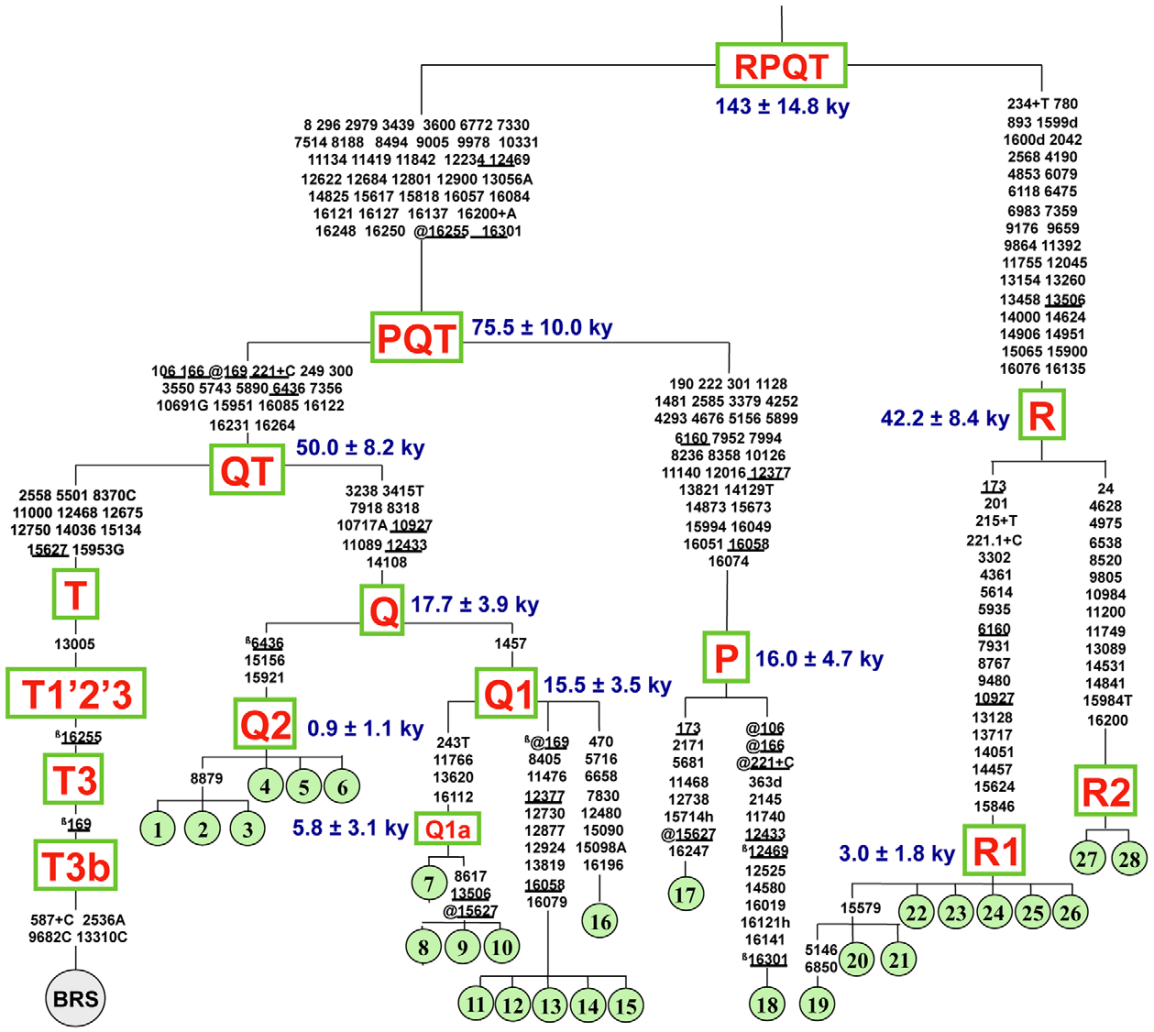
Tree of complete bovine mtDNA sequences. This tree, built and rooted as previously described by Achilli et al. 2008 [9] illustrates the relationships between the common haplogroup T represented by BRS and the rare mtDNAs belonging to haplogroups P, Q and R. Shown divergence times are those obtained using ML as reported in Table 3. Mutations are shown on the branches and are numbered according to the BRS; they are transitions unless a base is explicitly indicated; suffixes indicate transversions (to A, G, C, or T) or indels (+, d) and should be read as if the BRS was an artificial root. Recurrent mutations are underlined, and true back mutations with respect to evolutionary direction are prefixed with the superscript β (beta) in addition (which is thus in alternation with prefix @ on the path between the overall root and BRS). Note that the reconstruction of recurrent mutations in the control region is ambiguous in a number of cases. Heteroplasmy is marked with a suffix (h). The numbering of sequences is the same as in Figure 1.

ID numbers correspond to the numbers in Figures 1 and 2.

#### Haplogroup Q

The Q branch encompasses a total of 16 mtDNAs from five Italian breeds (Cabannina, Chianina, Grey Alpine, Italian Red Pied, and Romagnola). Complete sequence analysis revealed six different sequence types (haplotypes) clustering into the two distinct sub-haplogroups, Q1 (ten subjects; four haplotypes) and Q2 (six subjects; two haplotypes) (Figure 2). In other words, most of the six haplotypes were found in more than one animal. As expected, subjects with an identical sequence were generally from the same breed, but this was not always the case. For instance, the same haplotype was shared by two Grey Alpine (#11–12) and three Cabannina (#13–15) mtDNAs, and another haplotype was found in two Romagnola (#4–5) and one Chianina (#6), thus suggesting gene flow of maternal lineages between these breeds or their ancestors.

The maximum-likelihood (ML) divergence based on the complete mtDNA sequence for the entire Q haplogroup was 0.00036±0.00008 substitutions per site (Figure 1 and Table 3), and corresponded to a divergence time of 17.7±3.9 ky according to the mutation rate proposed by Achilli et al. 2008 [9]. The ML divergence for Q1 (0.00032±0.00007) was not much lower than that of the entire Q, and corresponded to 15.5±3.5 ky. In contrast, the extent of sequence variation within Q2 was extremely low resulting in a coalescence time of only 0.9±1.1 ky. These divergence ages were overall confirmed when the average distance of the haplotypes from the root of Q, Q1 and Q2 (ρ-statistics) was computed (Table 3).

**Table 3:** MtDNA haplogroup divergence values and time estimates obtained by using Maximum Likelihood (ML) and ρ statistics.

		Maximum Likelihood				ρ b Statistics			
Haplogroups/	No.of	Substitutions	S.E.	T	±ΔT	ρ	σ	T	±ΔT
Subhaplogroups	mtDNAs a	per site		(ky) c	(ky)			(ky) c	(ky)
RPQT	29	0.00292	0.00030	143.0	14.8	42.590	4.420	135.1	14.0
>PQT	19	0.00154	0.00020	75.5	10.0	21.000	3.559	66.6	11.3
>>QT	17	0.00102	0.00017	50.0	8.2	14.235	3.079	45.2	9.8
>>>Q	16	0.00036	0.00008	17.7	3.9	5.250	1.284	16.7	4.1
>>>>Q2	6	0.00002	0.00002	0.9	1.1	0.500	0.500	1.6	1.6
>>>>Q1	10	0.00032	0.00007	15.5	3.5	5.900	1.552	18.7	4.9
>>>>>Q1a	4	0.00012	0.00006	5.8	3.1	2.250	1.299	7.1	4.1
>>P	2	0.00033	0.00010	16.0	4.7	5.500	1.658	17.4	5.3
>R	10	0.00086	0.00017	42.2	8.4	13.900	3.084	44.1	9.8
>>R1	8	0.00006	0.00004	3.0	1.8	0.625	0.415	2.0	1.3
>>R2	2	n.a.	n.a.	n.a.	n.a.	n.a.	n.a.	n.a.	n.a.

a These comprise the sequences shown in Figures 1 and 2. Additional information regarding each mtDNA sequence is provided in Table 2.

b Average number of base substitutions in the mtDNA coding region (between nps 364 and 15791) from the ancestral sequence type. For haplogroups RPQT, PQT, QT, Q, Q1 and R, the contribution to ρ from each subclade is weighted based on their individual standard errors.

c Estimate of the time to the most recent common ancestor of each clade, using a mutation rate estimate of 3,172 years per substitution in the whole coding region (15,428 bp) [9].

#### Haplogroup P

The complete sequence of the mitochondrial genome from a Mesolithic wild aurochs became recently available [27], so it was possible to include it in our phylogeny (#18 in Figures 1 and 2) along with the only available modern complete P sequence (#17) [9]. The ancient DNA sequence derives from a humerus bone sample excavated from a cave in Derbyshire (UK), and was classified as *Bos primigenius* based on radiocarbon dating to 6,738±68 calibrated years BP [5]. The availability of two complete P sequences allowed the evaluation of the internal divergence of P (0.00033±0.00010 substitutions per site), which corresponds to a coalescence time of 16.0±4.7 ky (Figure 2). In addition, it was also possible to better assess the split time of the P branch from the sister branch QT (75.5±10 ky). However, both figures should be corrected to compensate for the fact that the Mesolithic aurochs sample died 6,738 years BP. Using the coding region mutation rate of 3,172 years [9] the adjusted divergence time for the P node should be moved up to approximately 20 ky. It is likely that the coalescence time of the P node will be soon further reassessed, not only by adding complete mtDNA sequences from additional ancient aurochs remains, but also from additional modern samples, at least four reported, whose mtDNA sequence data clearly indicate an affiliation within P [28]–[30].

#### Haplogroup R

Overall, haplogroup R comprises ten mtDNAs from four breeds (Agerolese, Cinisara, Romagnola and Marchigiana). The addition of six new sequences to those previously reported [10] allowed the dissection of R into two subhaplogroups, R1 and R2. The first encompasses eight mtDNAs, and contains a sub-branch defined by a coding region mutation at np 15579 separating the subjects previously described (Agerolese #19 and Cinisara #20–21) from the five new identical mtDNAs found in the Romagnola breed (#22–26). In addition, two other coding-region mutations (nps 5146 and 6850) distinguish the Agerolese from the two Cinisara. In contrast, R2 comprises only two mtDNAs with an identical haplotype, one from a Romagnola (#27) and one from a Marchigiana (#28). The overall divergence time of haplogroup R is 42.2±8.4 ky, thus confirming the ancient split between R1 and R2 [10]. In contrast, the radiation time of R1 estimated both with ML (3.0±1.8 ky) and ρ-statistics (2.0±1.3 ky) appears to be rather low.

## Discussion

Our survey of 25 Italian and twelve European taurine breeds confirmed the predominance of T mtDNAs in Europe, as expected. Yet two of the three non-T haplogroups, namely Q and R, made up 1.4% of our sample. Despite their frequencies are similarly low (0.8% and 0.6%, respectively), the distinct features of Q and R appear to indicate that their ancestral homelands and genetic history are different. To properly evaluate this issue, we have taken into account not only the complete mtDNA sequences that we report in this study, but also all the control-region data and the partial coding sequences available in the literature or posted in GenBank.

### Extensive Similarities between Haplogroups Q and T

This study provides a rather high number (16) of complete mtDNA sequences belonging to Q1 and Q2. These sequences depart from a central Q node (Figures 1 and 2) by 5.2–5.6 substitutions in the coding region (15,428 bp), corresponding to an estimated divergence time of 16–18 ky (Table 3) when using the evolutionary rate estimate of 2.043±0.099×10^−8^ base substitutions per nucleotide per year [9]. A comparable coalescence time (15–19 ky) characterizes sub-haplogroup Q1, which harbors three very deep branches departing directly from its nodal haplotype. One of these, showing some internal diversity (two haplotypes), was here termed Q1a (Figure 2).

The average sequence diversity and divergence time of Q are similar to those reported for haplogroup T (5.1±1.00 substitutions; 16.0±3.2 ky) [9]. Moreover, Q and T are the closest taurine branches in the tree (Figure 2) with a divergence time of about 40–50 ky (Table 3). Therefore, among all bovine haplogroups, haplogroups Q and T are those with the highest chance of having been present until recently in the same aurochs population, or at least in the same geographical area.

The finding that both Q and Q1 nodes coalesce prior to 10 kya, the upper limit for the domestication of *B. primigenius*, suggests that at least two different founder mtDNA haplotypes, one for Q1 and one for Q2 were acquired from wild aurochsen. This constitutes another similarity with the macro-haplogroup T, for which several founder haplotypes – at least one each for T1, T2, T3, T4 and T5 – have been proposed. However, one of our results seems at odds with the supposed similarity between Q and T. Indeed, all sub-haplogroups of T (possibly except T4) are geographically widespread, while we have detected Q mtDNAs only in Italian taurine breeds. One possibility is that Q is rare, for instance like sub-haplogroup T5, thus its geographical distribution needs to be assessed in greater detail.

To address this issue, we searched for the control-region mutational motif of Q (169-15953G-16255) in the whole GenBank dataset of bovine mtDNA sequences from both modern and ancient samples (a total of 4,770 sequences at the time of our analysis). Overall, we detected seven mtDNAs with such a motif, three from ancient bone specimens and four from modern populations. The ancient samples are all from distant Neolithic archaeological sites, one in Germany [26], one in eastern Thrace [31], [32] and one in France [26]. As for the modern samples, two are from local breeds of southwestern China [33] one from Turkey (GenBank EF126311), and one from Portugal [34]. Two other mtDNAs from China, for which instead only the *cytb* gene sequence was determined [35] were found to harbor the mutational motif 15134-15627, which is typical of Q (Figure 2).

In addition to these nine likely Q sequences, we also identified 70 modern and ancient mtDNAs, from a wide range of breeds and geographical areas, which potentially cluster within haplogroup Q. Their haplogroup affiliation is ambiguous because they are predominantly short control-region sequences that do not encompass np 15953, but the presence of the 16255 mutation places them outside of the common haplogroup T3, and its derivative T4 [9]. Moreover, they do not harbor the diagnostic mutations of haplogroups T1 (16113), T2 (16057C, 16185) or T5 (163), or those of haplogroups P and R. Additional sequencing of these mtDNA is necessary to exclude their affiliation within the rare haplogroup T1′2′3 (only two out of 1,747 in our sample), or even to a still unknown T haplogroup, but it is likely that most of these, if not all, are true members of Q. This implies that the distribution range of haplogroup Q covers at least several European countries, Egypt, Turkey and China, which is another major similarity between haplogroups Q and T. Therefore, a parallel history can be envisioned for the two haplogroups, with Q representing an additional minor lineage that was domesticated in the Near East and later spread with human migrations and trades.

### The Enigmatic Geographical Distribution of Haplogroup R

**Figure 3:**
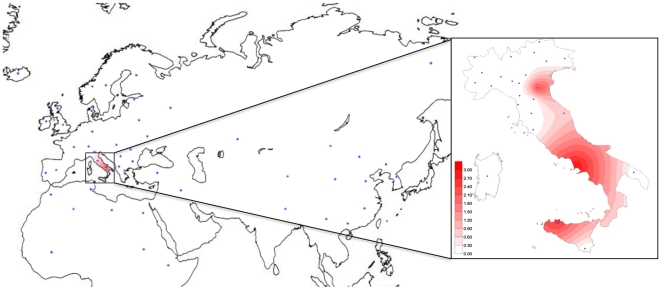
Spatial frequency distribution (%) of haplogroup R. The dots indicate the geographical location of the populations included in the survey. Population samples and corresponding frequency values are listed in Table S2. This frequency map was obtained using Surfer version 6.04 (Golden Software, Inc.), with the Kriging procedure, and estimates at each grid node were inferred by considering the entire data set.

What we found intriguing about haplogroup R is its occurrence only in Italian breeds (Table 1). It is true that our dataset is biased towards Italian breeds and that at a first glance the distribution of R resembles that of haplogroup Q, but this turned out not to be the case when we merged our data with those obtained from an extensive survey of the whole set of bovine mtDNA sequences available in GenBank and the literature. Unlike haplogroup Q that is defined by a simple control-region motif comprising only three mutations, of which one –169 – is unstable, the control-region motif of R harbors 27 distinguishing mutations relative to BRS (Figure 2), thus allowing the easy detection of its members, even by partial control-region sequencing. Thus, within 4,675 *B. taurus* sequences and 95 *B. primigenius* sequences (including complete mitochondrial genomes, control regions, *cytb* or other sequences) deposited in GenBank, none can be attributed to haplogroup R. The uniqueness of haplogroup R is best illustrated in Figure 3 and Table S2 that show its frequency distribution in old World cattle populations. The geographical distribution of haplogroup R shares no commonalities with that of haplogroup T and its sub-haplogroups or with that of haplogroup Q either. To date Italy is the only place where haplogroup R, the most divergent of taurine haplogroups in the phylogeny (Figure 1), has been detected.

### Is Haplogroup R Derived from a European Population of Aurochs?

Haplogroup R in Italy is as rare as Q, but a significant difference is that R is not – or at least not yet – found anywhere else in the world. This means that a domestication event in the Middle East, followed by dispersal in Europe, Africa and Eastern Asia along with Neolithic farmers and pastoralists - a probable scenario for the occurrence of Q mtDNAs in modern breeds – is unlikely for haplogroup R. Moreover, the extreme time divergence of the RPQT node (∼143 ky), almost twice that of the PQT node (∼76 ky), is compatible with the possibility that haplogroup R derives from herds of aurochs that were geographically distinct and distant from those of southwest Asia. Thus, an origin of R from European aurochs is a plausible scenario. However, if so, why is haplogroup R found only in Italy and not elsewhere in Europe?

One possible explanation requires taking into account the climatic changes that affected Europe at the end of the Pleistocene. Italy was one of the European refugia during the Last Glacial Maximum (LGM) (centered at about 21 kya), but in contrast to other refugia of southern Europe, the post-glacial expansion of its refugial populations to the North was restricted by the Alps [36]. Thus, if haplogroup R was confined to the Italian refuge area during the LGM, it might have been restricted there also afterward [10], as probably happened to the Italian-specific mtDNA haplogroups of other mammalian species, e.g. roe deer, boar and red squirrel [21], [37], [38].

Moreover, phylogenetic analyses show that haplogroup R is made up by two lineages, R1 and R2, which diverge from each other (about 42 ky), almost as much as the QT node (Figures 1 and 2). Therefore, at least two wild female aurochs were able to pass their mtDNA across generations to modern Italian cattle. How and when this event occurred?

Two scenarios, not necessarily mutually exclusive, can be envisioned to explain haplogroup R in Italian taurine breeds. The first is that R1 and R2 mtDNAs are due to sporadic interbreeding events (naturally occurring and/or man mediated) between wild aurochs cows and domestic bulls, followed by incorporation of the offspring in the domestic herds by Neolithic or post-Neolithic pastoralists. The second possibility is that, somewhere in Italy, there was a minor and independent event of *B. primigenius* domestication. Both scenarios are compatible with the coalescence time (2–3 ky) of the R1 sequences (Figure 2). Taking into account that five of the nine R1 sequences (# 22–26 in Fig. 2) are identical at the root of R1, such coalescence time might be underestimated. However, even if we include only one of these identical sequences in the calculations and exclude all the others, the average divergence time of R1 is only pushed back to about 4–5 ky

It is interesting that Virgil in 29 BC [39] describes a plague affecting domestic cattle in Northern Italy and the use of wild aurochs (“uris”) instead of oxen to pull a ritual carriage during a feast to celebrate Juno. Thus, the taming of wild aurochs in the Virgil's report seems almost common place, certainly much easier than the taming of the impressive beasts described by Caesar in the Black Forest of southern Germany [40]. Maybe aurochs in Italy were smaller and more docile than their northern European counterparts. We have found haplogroup R mtDNAs in breeds that come from Campania (Agerolese) and Sicily (Cinisara), among others. This does not necessarily mean that aurochsen with R mDNAs were domesticated there, yet it is intriguing that some authors point out how aurochsen in Sicily became smaller in size after the disappearance of the land bridge with mainland Italy, which means they likely became much more amenable to taming [41], [42].

An additional argument in favor of the possibility of a local domestication event of European aurochs is provided by recent mtDNA data concerning the domestication of other mammalian species, in particular of those where the wild animals are not extinct, the domestic pig and the wild boar [21]. Modern European pig breeds are grouped in a large mtDNA cluster that bears no affinity to wild boar lineages of the Middle East, which means that, if domesticated lineages came to Europe with farmers from the Near East, they left no trace in modern European breeds. Besides, network analysis revealed two core lineages in Europe, with star-like patterns suggestive of a recent population expansion, like for cattle, except that two such pig core haplotypes are found only in Europe. In short, there is good evidence to support the domestication of at least two wild boar mtDNA lineages in Europe.

An interesting implication of the study on pig domestication is that multiple domestication events cannot be ruled out even for those species where the wild progenitors are extinct, such as *B. primigenius* in Europe. A recent study carried out with 37K SNPs on modern cattle breeds [43] shows that *Bos taurus* has a remarkable nuclear genetic diversity and had a large ancestral population until recently, before fragmentation during breed formation. This is in contrast with the low level of mtDNA diversity observed in this species, largely predominated by the T haplogroup. The genetic contribution of different aurochs populations may have really played a role in shaping the existing diversity by events of interbreeding and/or domestication at different times and locations.

Only sequencing of DNA from the remains of ancient aurochs, throughout their original distribution range, will shed light on the issue of additional domestication events. However, scenarios of multiple domestication events scattered across different geographical areas applicable to a number of plant and animal species [44] may be extended to cattle. We think that our data are not incompatible with the scenario that one such event, marked by haplogroup R, might have happened in Italy in rather recent times.

## Materials and Methods

### Ethics statement

All experimental procedures were reviewed and approved by the Animal Research Ethics Committee of the University of Pavia, Prot. 2/2007 (April 17^th^, 2007), in accordance with the European Union Directive 86/609.

### Samples

A set of 1,747 animals was analyzed, encompassing 25 Italian breeds and twelve other European breeds. This figure includes 1,128 new subjects and 619 animals (22 Italian and 4 European breeds) previously reported [10], extending breed representation to include three novel breeds from Italy (Marchigiana, Reggiana, Pettiazza) and eight from Europe (Blacksided Trondheim, Bulgarian Grey, Eastern Finn Cattle, Grey Steppe, Jersey, Swedish Red Polled, Vestland Red Polled, Telemark). DNAs were purified from either peripheral blood or platelets according to standard methods. All experimental procedures were reviewed and approved by the Animal Research Ethics Committee of the University of Pavia, in accordance with the European Union Directive 86/609.

### Sequences analysis of the mtDNA control region

For all animals, a PCR fragment of 1138 bp encompassing the mtDNA control region (np 15718-517) was sequenced using the oligonucleotide 15757for, 5′ CCCCAAAGCTGAAGTTCTAT 3′, as previously described [9]. Reads covered at least 730 bp, from np 15823 to np 215 (Table S1). Sequences were aligned to the Bovine Reference Sequence (BRS) using the Sequencher software (Gene Codes Corporation) and the identified mutational motifs [10] were used to classify mtDNAs within haplogroups.

### Sequencing of entire mitochondrial genomes

The entire sequence of all mtDNAs harboring Q and R control-region mutational motifs was determined as previously reported [9]. In brief, a set of 11 overlapping PCR fragments covering the entire mtDNA genome was produced and sequenced by standard dideoxysequencing with 32 nested oligonucleotides. To derive individual sequences, raw sequence data were grouped into mtDNA genome contigs and compared to the BRS [45].

### MtDNA phylogeny and time estimates

A Mid-rooting point has been used for the tree of Figure 1, while that of Figure 2 was built and rooted by using a *Bos grunniens* (yak) and a *Bison bison* (American bison) mitochondrial genome, as previously described [9]. The evolutionary distances were computed using the Maximum Likelihood method together with averaged distance (ρ) of the haplotypes within a clade from the respective root haplotype, accompanied by a heuristic estimate of SE (σ). All positions containing gaps and ambiguous data were eliminated from the dataset. Estimate of the time to the most recent common ancestor for each cluster was calculated using a corrected age estimate of about 3,172 years per substitution in the whole coding region (15,428 bp) [9].

## Supporting Information

Table S1 MtDNA control‐region haplotypes observed in the 1,747 taurine samples included in this study.
(XLS)

Table S2 Distribution of haplogroup R in bovine populations.
(DOC)
