# Supplementary material for: The Enigmatic Origin of Bovine mtDNA Haplogroup R: Sporadic Interbreeding or an Independent Event of Bos primigenius Domestication in Italy?
Source: PLoS One. 2010 Dec 28;5(12):e15760. doi: 10.1371/journal.pone.0015760 (PMC3011016; doi:10.1371/journal.pone.0015760)
Supplement: Table S2 — Distribution of haplogroup R in bovine populations. (DOC) [file pone.0015760.s002.doc]

**Table S2** Distribution of Haplogroup R in Bovine Populations

| **Region, Country** | **Sample size** | **Haplogroup R** | | **References** |
| --- | --- | --- | --- | --- |
|  |  | **No of subjects** | **%** |  |
| ***Western Europe*** |  |  |  |  |
| Denmark | 18 | 0 | - | [18] |
| England | 16 | 0 | - | [7, 13, 24, 35] |
| Finland | 54 | 0 | - | [1, 18] |
| France | 109 | 0 | - | [1, 5, 6, 7, 12, 13, 24, 35] |
| Germany | 47 | 0 | - | [4, 6, 21, 31, 33, 35] |
| Iceland | 23 | 0 | - | [25, 35] |
| Ireland | 32 | 0 | - | [6, 25, 35] |
| Italy North | 646 | 0 | - | [1, 2, 3, 5, 30] |
| Italy, Apulia | 112 | 0 | - | [1, 2, 3, 30] |
| Italy, Campania | 33 | 1 | 3.0 | [1, 3] |
| Italy, Marche | 149 | 1 | 0.7 | [1, 5] |
| Italy, Romagna | 253 | 6 | 2.4 | [1, 3, 5, 30, 35] |
| Italy, Sardinia | 36 | 0 | - | [1, 2] |
| Italy, Sicily North | 81 | 2 | 2.5 | [1, 2, 3, 5, 30] |
| Italy, Sicily South | 22 | 0 | - | [1, 2, 3, 30] |
| Italy, Tuscany | 463 | 0 | - | [1, 2, 3, 5, 30] |
| Norway | 73 | 0 | - | [1, 25, 35] |
| Portugal | 521 | 0 | - | [5, 10, 14] |
| Scotland | 44 | 0 | - | [7, 24, 35 ] |
| Spain | 755 | 0 | - | [2, 4, 5, 9, 14, 28, 29 ] |
| Sweden | 53 | 0 | - | [1, 18] |
| Switzerland | 27 | 0 | - | [1, 16, 32] |
| ***Eastern Europe*** |  |  |  |  |
| Albania | 8 | 0 | - | [5] |
| Bulgaria | 38 | 0 | - | [1, 5] |
| Byelorussia | 8 | 0 | - | [18] |
| Estonia | 10 | 0 | - | [18] |
| Greece | 48 | 0 | - | [2, 5, 30, 35] |
| Hungary, Slovenia, Slovakia | 54 | 0 | - | [1, 5, 6, 13] |
| Romania | 10 | 0 | - | [5] |
| European Russia: Kirov, Nizhegorov, Perm, Mari regions, Yaroslavl; Georgia | 59 | 0 | - | [6, 18] |
| Russia, Northeast Siberia | 24 | 0 | - | [18] |
| Serbia | 29 | 0 | - | [5, 18] |
| Ukraine | 30 | 0 | - | [5, 18] |
| ***Middle East*** |  |  |  |  |
| Iran, Iraq, Syria | 55 | 0 | - | [2, 12, 35] |
| Turkey | 66 | 0 | - | [5, 35] |
| ***Central Asia*** |  |  |  |  |
| Kazakshtan, Uzbekistan | 9 | 0 | - | [18] |
| ***Eastern Asia*** |  |  |  |  |
| China: Anhui, Hubei, Fujian, Jiangxi, Zhejiang | 100 | 0 | - | [17, 22, 23] |
| China: Gansu, Shaanxi, Sichuan | 144 | 0 | - | [8, 17, 22, 23] |
| China: Guangdong, Guangxi, Guizhou, Hainan, Hunan | 151 | 0 | - | [17, 23] |
| China: Yunnan | 91 | 0 | - | [15, 17, 22] |
| China: Henan, Shandong, Shanxi | 65 | 0 | - | [22, 23] |
| China: Inner Mongolia | 18 | 0 | - | [17, 23] |
| China: Jilin, Liaoning | 43 | 0 | - | [17, 23] |
| China: Tibet, Xinjiang | 52 | 0 | - | [17, 23] |
| Japan | 32 | 0 | - | [26] |
| Korea | 46 | 0 | - | [19, 20, 27] |
| Mongolia | 44 | 0 | - | [27] |
| Nepal | 13 | 0 | - | [34] |
| ***Africa*** |  |  |  |  |
| Algeria | 22 | 0 | - | [5] |
| Egypt | 32 | 0 | - | [5, 35] |
| Ethiopia | 11 | 0 | - | [11] |
| Libya | 24 | 0 | - | [5] |
| Morocco | 58 | 0 | - | [5] |
| Sudan | 24 | 0 | - | [5, 35] |
| Tunisia | 13 | 0 | - | [5] |
| West Africa | 68 | 0 | - | [7, 12, 24, 35] |
| ***TOTAL*** | 5066 | 10 | 0.2 |  |

**References**

1. This study.

2. Achilli A, Olivieri A, Pellecchia M, Uboldi C, Colli L, et al. (2008) Mitochondrial genomes of extinct aurochs survive in domestic cattle. Curr Biol 18: R157-R158.

3. Achilli A, Bonfiglio S, Olivieri A, Malusà A, Pala M, et al. (2009) The multifaceted origin of taurine cattle reflected by the mitochondrial genome. PLoS ONE 4: e5753.

4. [Anderung C](http://www.ncbi.nlm.nih.gov/pubmed?term="Anderung C"%5BAuthor%5D), [Bouwman A](http://www.ncbi.nlm.nih.gov/pubmed?term="Bouwman A"%5BAuthor%5D), [Persson P](http://www.ncbi.nlm.nih.gov/pubmed?term="Persson P"%5BAuthor%5D), [Carretero JM](http://www.ncbi.nlm.nih.gov/pubmed?term="Carretero JM"%5BAuthor%5D), [Ortega AI](http://www.ncbi.nlm.nih.gov/pubmed?term="Ortega AI"%5BAuthor%5D), et al. (2005) Prehistoric contacts over the Straits of Gibraltar indicated by genetic analysis of Iberian Bronze Age cattle. [Proc Natl Acad Sci USA](javascript:AL_get(this, 'jour', 'Proc Natl Acad Sci U S A.');) 102:8431-8435.

5. Beja-Pereira A, Caramelli D, Lalueza-Fox C, Vernesi C, Ferrand N, et al. (2006) The origin of European cattle: evidence from modern and ancient DNA. Proc Natl Acad Sci USA 103: 8113–8118.

6. Bollongino R, Edwards CJ, Alt KW, Burger J, Bradley DG (2006) Early history of European domestic cattle as revealed by ancient DNA. Biol Lett 2: 155–159.

7. Bradley DG, MacHugh DE, Cunningham P, Loftus RT (1996) Mitochondrial diversity and the origins of African and European cattle. Proc Natl Acad Sci USA 93: 5131–5135.

8. [Chen SY](http://www.ncbi.nlm.nih.gov/pubmed?term="Chen SY"%5BAuthor%5D), [Liu YP](http://www.ncbi.nlm.nih.gov/pubmed?term="Liu YP"%5BAuthor%5D), [Wang W](http://www.ncbi.nlm.nih.gov/pubmed?term="Wang W"%5BAuthor%5D), [Gao CZ](http://www.ncbi.nlm.nih.gov/pubmed?term="Gao CZ"%5BAuthor%5D), [Yao YG](http://www.ncbi.nlm.nih.gov/pubmed?term="Yao YG"%5BAuthor%5D), [et](http://www.ncbi.nlm.nih.gov/pubmed?term="Lai SJ"%5BAuthor%5D) al. (2008) Dissecting the matrilineal components of Tongjiang cattle from southwest China. Biochem Genet 46: 206-215.

9. [Cortés O](http://www.ncbi.nlm.nih.gov/pubmed?term="Cortés O"%5BAuthor%5D), [Tupac-Yupanqui I](http://www.ncbi.nlm.nih.gov/pubmed?term="Tupac-Yupanqui I"%5BAuthor%5D), [Dunner S](http://www.ncbi.nlm.nih.gov/pubmed?term="Dunner S"%5BAuthor%5D), [García-Atance MA](http://www.ncbi.nlm.nih.gov/pubmed?term="García-Atance MA"%5BAuthor%5D), [García D](http://www.ncbi.nlm.nih.gov/pubmed?term="García D"%5BAuthor%5D), et al. (2008) Ancestral matrilineages and mitochondrial DNA diversity of the Lidia cattle breed. Anim Genet 39: 649-654.

10. [Cymbron T](http://www.ncbi.nlm.nih.gov/pubmed?term="Cymbron T"%5BAuthor%5D), [Loftus RT](http://www.ncbi.nlm.nih.gov/pubmed?term="Loftus RT"%5BAuthor%5D), [Malheiro MI](http://www.ncbi.nlm.nih.gov/pubmed?term="Malheiro MI"%5BAuthor%5D), [Bradley DG](http://www.ncbi.nlm.nih.gov/pubmed?term="Bradley DG"%5BAuthor%5D) (1999) Mitochondrial sequence variation suggests an African influence in Portuguese cattle. Proc Biol Sci 266: 597-603.

11. [Dadi H](http://www.ncbi.nlm.nih.gov/pubmed?term="Dadi H"%5BAuthor%5D), [Tibbo M](http://www.ncbi.nlm.nih.gov/pubmed?term="Tibbo M"%5BAuthor%5D), [Takahashi Y](http://www.ncbi.nlm.nih.gov/pubmed?term="Takahashi Y"%5BAuthor%5D), [Nomura K](http://www.ncbi.nlm.nih.gov/pubmed?term="Nomura K"%5BAuthor%5D), [Hanada H](http://www.ncbi.nlm.nih.gov/pubmed?term="Hanada H"%5BAuthor%5D), et al. (2009) Variation in mitochondrial DNA and maternal genetic ancestry of Ethiopian cattle populations. Anim Genet 40: 556-559.

12. Edwards CJ, MacHugh DE, Dobney KM , Martin L, Russell N, et al. (2004) Ancient DNA analysis of 101 cattle remains: limits and prospects. J Archaeol Sci 31: 695-710.

13. Edwards CJ, Bollongino R, Scheu A, Chamberlain A, Tresset A, et al. (2007) Mitochondrial DNA analysis shows a Near Eastern Neolithic origin for domestic cattle and no indication of domestication of European aurochs. Proc Biol Sci 274: 1377–1385.

14. Ginja C, Penedo MC, Melucci L, Quiroz J, Martínez López OR, et al. (2010) Origins and genetic diversity of New World Creole cattle: inferences from mitochondrial and Y chromosome polymorphisms. Anim Genet 41: 128-141.

15. [Gou X](http://www.ncbi.nlm.nih.gov/pubmed?term="Gou X"%5BAuthor%5D), [Wang Y](http://www.ncbi.nlm.nih.gov/pubmed?term="Wang Y"%5BAuthor%5D), [Yang S](http://www.ncbi.nlm.nih.gov/pubmed?term="Yang S"%5BAuthor%5D), [Deng W](http://www.ncbi.nlm.nih.gov/pubmed?term="Deng W"%5BAuthor%5D), [Mao H](http://www.ncbi.nlm.nih.gov/pubmed?term="Mao H"%5BAuthor%5D) (2010) Genetic diversity and origin of Gayal and cattle in Yunnan revealed by mtDNA control region and SRY gene sequence variation. J Anim Breed Genet 127: 154-160.

16. [Hiendleder S](http://www.ncbi.nlm.nih.gov/pubmed?term="Hiendleder S"%5BAuthor%5D), [Lewalski H](http://www.ncbi.nlm.nih.gov/pubmed?term="Lewalski H"%5BAuthor%5D), [Janke A](http://www.ncbi.nlm.nih.gov/pubmed?term="Janke A"%5BAuthor%5D) (2008) Complete mitochondrial genomes of *Bos taurus* and *Bos indicus* provide new insights into intra-species variation, taxonomy and domestication. Cytogenet Genome Res 120: 150-156.

17. [Jia S](http://www.ncbi.nlm.nih.gov/pubmed?term="Jia S"%5BAuthor%5D), [Zhou Y](http://www.ncbi.nlm.nih.gov/pubmed?term="Zhou Y"%5BAuthor%5D), [Lei C](http://www.ncbi.nlm.nih.gov/pubmed?term="Lei C"%5BAuthor%5D), [Yao R](http://www.ncbi.nlm.nih.gov/pubmed?term="Yao R"%5BAuthor%5D), [Zhang Z](http://www.ncbi.nlm.nih.gov/pubmed?term="Zhang Z"%5BAuthor%5D), et al. (2010) A new insight into cattle's maternal origin in six Asian countries. J Genet Genomics 37: 173-180.

18. [Kantanen J](http://www.ncbi.nlm.nih.gov/pubmed?term="Kantanen J"%5BAuthor%5D), [Edwards CJ](http://www.ncbi.nlm.nih.gov/pubmed?term="Edwards CJ"%5BAuthor%5D), [Bradley DG](http://www.ncbi.nlm.nih.gov/pubmed?term="Bradley DG"%5BAuthor%5D), [Viinalass H](http://www.ncbi.nlm.nih.gov/pubmed?term="Viinalass H"%5BAuthor%5D), [Thessler S](http://www.ncbi.nlm.nih.gov/pubmed?term="Thessler S"%5BAuthor%5D), et al. (2009) Maternal and paternal genealogy of Eurasian taurine cattle (*Bos taurus*). Heredity 103: 404-415.

19. [Kim KI](http://www.ncbi.nlm.nih.gov/pubmed?term="Kim KI"%5BAuthor%5D), [Lee JH](http://www.ncbi.nlm.nih.gov/pubmed?term="Lee JH"%5BAuthor%5D), [Lee SS](http://www.ncbi.nlm.nih.gov/pubmed?term="Lee SS"%5BAuthor%5D), [Yang YH](http://www.ncbi.nlm.nih.gov/pubmed?term="Yang YH"%5BAuthor%5D) (2003) Phylogenetic relationships of Northeast Asian cattle to other cattle populations determined using mitochondrial DNA D-loop sequence polymorphism. Biochem Genet 41: 91-98.

20. [Kim JH](http://www.ncbi.nlm.nih.gov/pubmed?term="Kim JH"%5BAuthor%5D), [Oh JH](http://www.ncbi.nlm.nih.gov/pubmed?term="Oh JH"%5BAuthor%5D), [Song JH](http://www.ncbi.nlm.nih.gov/pubmed?term="Song JH"%5BAuthor%5D), [Jeon JT](http://www.ncbi.nlm.nih.gov/pubmed?term="Jeon JT"%5BAuthor%5D), [Han SH](http://www.ncbi.nlm.nih.gov/pubmed?term="Han SH"%5BAuthor%5D), et al. (2005) Molecular genetic analysis of ancient cattle bones excavated from archaeological sites in Jeju, Korea. Mol Cells 20: 325-330.

21. [Kühn R](http://www.ncbi.nlm.nih.gov/pubmed?term="Kühn R"%5BAuthor%5D), [Ludt C](http://www.ncbi.nlm.nih.gov/pubmed?term="Ludt C"%5BAuthor%5D), [Manhart H](http://www.ncbi.nlm.nih.gov/pubmed?term="Manhart H"%5BAuthor%5D), [Peters J](http://www.ncbi.nlm.nih.gov/pubmed?term="Peters J"%5BAuthor%5D), [Neumair E](http://www.ncbi.nlm.nih.gov/pubmed?term="Neumair E"%5BAuthor%5D), [et](http://www.ncbi.nlm.nih.gov/pubmed?term="Rottmann O"%5BAuthor%5D) al. (2005) Close genetic relationship of early neolithic cattle from Ziegelberg (Freising, Germany) with modern breeds. J Anim Breed Genet 122: 36-44.

22. Lai S-J, Liu Y-P, Liu Y-X, Li X-W, Yao Y-G (2006) Genetic diversity and origin of Chinese cattle revealed by mtDNA D-loop sequence variation. Mol Phylogenet Evol 38: 146–154.

23. Lei CZ, Chen H, Zhang HC, Cai X, Liu RY, et al. (2006) Origin and phylogeographical structure of Chinese cattle. Anim Genet 37: 579-582.

24. Loftus RT, MacHugh DE, Bradley DG, Sharp PM, Cunningham P (1994) Evidence for two independent domestications of cattle. Proc Natl Acad Sci USA 91: 2757–2761.

25. [MacHugh DE](http://www.ncbi.nlm.nih.gov/pubmed?term="MacHugh DE"%5BAuthor%5D), [Troy CS](http://www.ncbi.nlm.nih.gov/pubmed?term="Troy CS"%5BAuthor%5D), [McCormick F](http://www.ncbi.nlm.nih.gov/pubmed?term="McCormick F"%5BAuthor%5D), [Olsaker I](http://www.ncbi.nlm.nih.gov/pubmed?term="Olsaker I"%5BAuthor%5D), [Eythórsdóttir E](http://www.ncbi.nlm.nih.gov/pubmed?term="Eythórsdóttir E"%5BAuthor%5D), [et](http://www.ncbi.nlm.nih.gov/pubmed?term="Bradley DG"%5BAuthor%5D) al. (1999) Early medieval cattle remains from a Scandinavian settlement in Dublin: genetic analysis and comparison with extant breeds. [Philos Trans R Soc Lond B Biol Sci](javascript:AL_get(this, 'jour', 'Philos Trans R Soc Lond B Biol Sci.');) 354: 99-108.

26. [Mannen H](http://www.ncbi.nlm.nih.gov/pubmed?term="Mannen H"%5BAuthor%5D), [Tsuji S](http://www.ncbi.nlm.nih.gov/pubmed?term="Tsuji S"%5BAuthor%5D), [Loftus RT](http://www.ncbi.nlm.nih.gov/pubmed?term="Loftus RT"%5BAuthor%5D), [Bradley DG](http://www.ncbi.nlm.nih.gov/pubmed?term="Bradley DG"%5BAuthor%5D) (1998) Mitochondrial DNA variation and evolution of Japanese black cattle (*Bos taurus*). Genetics 150: 1169-1175.

27. Mannen H, Kohno M, Nagata Y, Tsuji S, Bradley DG, et al. (2004) Independent mitochondrial origin and historical genetic differentiation in North Eastern Asian cattle. Mol Phylogenet Evol 32: 539–544.

28. [Miretti MM](http://www.ncbi.nlm.nih.gov/pubmed?term="Miretti MM"%5BAuthor%5D), [Dunner S](http://www.ncbi.nlm.nih.gov/pubmed?term="Dunner S"%5BAuthor%5D), [Naves M](http://www.ncbi.nlm.nih.gov/pubmed?term="Naves M"%5BAuthor%5D), [Contel EP](http://www.ncbi.nlm.nih.gov/pubmed?term="Contel EP"%5BAuthor%5D), [Ferro JA](http://www.ncbi.nlm.nih.gov/pubmed?term="Ferro JA"%5BAuthor%5D) (2004) Predominant African-derived mtDNA in Caribbean and Brazilian Creole cattle is also found in Spanish cattle (*Bos taurus*). J Hered 95: 450-453.

29. [Mirol PM](http://www.ncbi.nlm.nih.gov/pubmed?term="Mirol PM"%5BAuthor%5D), [Giovambattista G](http://www.ncbi.nlm.nih.gov/pubmed?term="Giovambattista G"%5BAuthor%5D), [Lirón JP](http://www.ncbi.nlm.nih.gov/pubmed?term="Lirón JP"%5BAuthor%5D), [Dulout FN](http://www.ncbi.nlm.nih.gov/pubmed?term="Dulout FN"%5BAuthor%5D) (2003) African and European mitochondrial haplotypes in South American Creole cattle. Heredity 91: 248-254

30. [Pellecchia M](http://www.ncbi.nlm.nih.gov/pubmed?term="Pellecchia M"%5BAuthor%5D), [Negrini R](http://www.ncbi.nlm.nih.gov/pubmed?term="Negrini R"%5BAuthor%5D), [Colli L](http://www.ncbi.nlm.nih.gov/pubmed?term="Colli L"%5BAuthor%5D), [Patrini M](http://www.ncbi.nlm.nih.gov/pubmed?term="Patrini M"%5BAuthor%5D), [Milanesi E](http://www.ncbi.nlm.nih.gov/pubmed?term="Milanesi E"%5BAuthor%5D), et al (2007) The mystery of Etruscan origins: novel clues from *Bos taurus* mitochondrial DNA. [Proc Biol Sci](javascript:AL_get(this, 'jour', 'Proc Biol Sci.');) 274: 1175-1179.

31. Scheu A, Hartz S, Schmölcke U, Tresset A, Burger J, et al. (2008) Ancient DNA provides no evidence for independent domestication of cattle in Mesolithic Rosenhof, Northern Germany. J Archaeol Sci 35: 1257-1264

32. [Schlumbaum A](http://www.ncbi.nlm.nih.gov/pubmed?term="Schlumbaum A"%5BAuthor%5D), [Turgay M](http://www.ncbi.nlm.nih.gov/pubmed?term="Turgay M"%5BAuthor%5D), [Schibler J](http://www.ncbi.nlm.nih.gov/pubmed?term="Schibler J"%5BAuthor%5D) (2006) Near East mtDNA haplotype variants in Roman cattle from Augusta Raurica, Switzerland, and in the Swiss Evolène breed. Anim Genet 37: 373-375.

33. [Steinborn R](http://www.ncbi.nlm.nih.gov/pubmed?term="Steinborn R"%5BAuthor%5D), [Müller M](http://www.ncbi.nlm.nih.gov/pubmed?term="Müller M"%5BAuthor%5D), [Brem G](http://www.ncbi.nlm.nih.gov/pubmed?term="Brem G"%5BAuthor%5D) (1998) Genetic variation in functionally important domains of the bovine mtDNA control region. Biochim Biophys Acta 1397: 295-304.

34. Takeda K, Satoh M, Neopane SP, Kuwar BS, Joshi HD, et al. (2004) Mitochondrial DNA analysis of Nepalese domestic dwarf cattle Lulu. Anim Sci J 75: 103-110.

35. Troy CS, MacHugh DE, Bailey JF, Magee DA, Loftus RT, et al. (2001) Genetic evidence for Near-Eastern origins of European cattle. Nature 410: 1088–1099.
